# Connecting the dots on health inequalities – a systematic review on the social determinants of health in Portugal

**DOI:** 10.1186/s12939-016-0314-z

**Published:** 2016-02-16

**Authors:** Inês Campos-Matos, Giuliano Russo, Julian Perelman

**Affiliations:** Instituto de Higiene e Medicina Tropical, Universidade NOVA de Lisboa, Lisbon, Portugal; Centro de Investigação em Saúde Pública, Lisbon, Portugal; Global Health and Tropical Medicine, Instituto de Higiene e Medicina Tropical, NOVA University of Lisbon, Lisbon, Portugal; Escola Nacional de Saúde Pública, Universidade NOVA de Lisboa, Lisbon, Portugal

**Keywords:** Portugal, Health inequalities, Social determinants of health, Socioeconomic factors

## Abstract

**Introduction:**

Health inequalities are recognised as a public health issue worldwide, but only a few countries have developed national strategies to monitor and reduce them. Despite its considerable health inequalities, Portugal seems to lack a systematic strategy to tackle them, possibly due to the absence of organised evidence on the issue. We performed a systematic review that aimed to describe the available evidence on social inequalities in health in Portugal, in order to contribute towards a comprehensive and focused strategy to tackle them.

**Methods:**

We followed the PRISMA guidelines and searched Scopus, Web of Science and PubMed for studies that looked at the association between a measure of socioeconomic status and a health outcome in the Portuguese resident population since the year 2000. We excluded health behaviours and healthcare use from our search. We performed a qualitative description of the results.

**Results:**

Seventy-one publications were selected, all reporting observational analyses, most of them using cross-sectional data. These publications showed strong evidence for health inequalities related to education and gender, chiefly for obesity, self-rated health and mental health.

**Conclusions:**

Analysis of the eligible publications showed that current research does not seem to have consistently covered the link between health and key Portuguese social problems. A strategy focusing on the monitoring of most prevalent diseases, most determining socioeconomic factors and vulnerable populations would be crucial to guide academic research in a country in which health inequalities are so ubiquitous and deeply rooted.

**Registration:**

This systematic review is not registered.

**Electronic supplementary material:**

The online version of this article (doi:10.1186/s12939-016-0314-z) contains supplementary material, which is available to authorized users.

## Introduction

Several individual socioeconomic characteristics such as occupation, employment or income, have been extensively shown to be associated with health outcomes [1]. The health inequalities that this creates have not gone unnoticed to academics and policy-makers, and a number of crucial publications, from the Black report in the UK [2] to the report of the Commission on the Social Determinants of Health by the World Health Organization (WHO) [1] have helped push this issue into the political agenda of several countries. As a result, numerous European countries like the UK, the Netherlands, Ireland, Sweden and Finland, have adopted and monitored policies to reduce health inequalities [3].

Portugal seems to have lagged behind in this issue, particularly in its political agenda. Pereira and Furtado (2011) noticed that despite it being one of the foundations on the legal documents regarding the Portuguese health system, interest in health equality has been practically non-existent in the country [3]. Two WHO reports on the Portuguese National Health Plan and on the Portuguese health system argued that health inequalities were an “important policy gap” [4] and recommended the “[promotion of] health policies targeting health gains and reduced health inequalities in all areas” [5].

There are indeed very good reasons to focus health inequalities in Portugal, as in 2011 it was one of the most unequal countries in the European Union, with the continent’s second highest Gini coefficient for disposable household income [6]. Not surprisingly, comparative analyses have shown that Portugal is also one of the European countries with the highest health inequalities. Mackenbach et al. (2008), for example, showed that Portugal had Europe’s highest education-related relative index of inequality in self-rated health (SRH) for both genders and in obesity for women [7].

Despite its high health inequalities and a constitutionally sanctioned commitment to health equity, Bago d’Uva argues that it is the absence of explicit and effective policies to tackle health inequalities allows them to persist so critically high [8]. Crucially, a real or perceived lack of evidence on health inequalities – its magnitude, causes, most affected areas, groups and diseases – limits the design and implementation of equity-oriented health policies.

This systematic review of the literature seeks to confront this absence, by aiming to describe the available evidence on social inequalities in health in Portugal. To the best of our knowledge, no similar review has been carried out in this context so far. This exercise has a dual purpose: to help define a research agenda on health inequalities in Portugal, by pointing out limitations in knowledge and to provide an evidence base to guide political decision-making. With this, we hope to offer a stepping-stone towards a comprehensive and adequately focused strategy to tackle health inequalities in Portugal.

## Methods

### Search strategy

A systematic review of published literature was conducted on health inequalities in Portugal. We followed the PRISMA statement to guide and report the review [9]. We searched for eligible articles in Portuguese and English using Scopus, Web of Science and PubMed. We focused on most recent work on the subject, limiting our search to publications after January 1^st^ 2000. Besides these database searches, we also scoped publications of recognized specialists in this field in Portugal and selected those that were relevant and met the outlined eligibility criteria. The detailed search strategy is outlined in the online Additional file 1.

### Study selection

We looked for studies that (i) analysed resident Portuguese population, (ii) looked for the association between a measure of socioeconomic status (SES) and health status, (iii) aimed to quantify the impact of SES on the outcome and (iv) in this quantification, controlled at least for age and gender as potential confounders. We followed the PROGRESS framework– standing for Place of residence, Race/ethnicity/culture/language, Occupation, Gender/sex, Religion, Education, Socioeconomic status and Social capital – to identify socioeconomic determinants of interest [10]. Both individual and contextual socioeconomic determinants were considered. Health outcomes were restricted to three types of indicators, following Blaxter’s classification [11]: medical, functional and subjective health. This excluded commonly mentioned mediators of the socioeconomic-health relationship, namely health related behaviours and healthcare use or access. We also excluded qualitative studies. Studies that analysed Portugal among other countries were not excluded, as long as a result for Portugal was presented. We included only studies that used data from the year 2000 onwards as to focus our study on contemporary issues.

The search and initial title screen were performed by one author, who identified relevant publications. The selected publications were independently analysed by two authors for compliance with inclusion and exclusion criteria. Any discrepancy was resolved in a panel discussion between the three authors.

### Data extraction

One author performed data extraction; uncertainties were resolved by a second author extracting the data independently. The following information was extracted from each publication: sample characteristics (sample size, geographic area and demographic characteristics), data source (for analyses based on previous surveys), exposure(s), outcome(s), study design (including sampling procedures), methods used in analysis, variables controlled for and main findings. Each publication was also assessed for strengths and limitations, considering the following items: sample size, sampling methods, control for confounders, appropriate measurement of variables, appropriate statistical analysis and possible sources of bias.

### Data presentation

The extracted data is summarized in the online Additional file 2. Data was first summarized through a table with a brief description of results according to combinations of SES and health variables. Subsequently, a diagram was drawn, where SES determinants were represented by circles proportional to the number of publications in which they were used. In this diagram, health outcomes were written in a font size also proportional to the number of publications in which each was used. Arrows connecting the two denote the strength of the associations found. Details on how this diagram was built are outlined in the online Additional file 3.

## Results

### Study selection

**Fig. 1:**
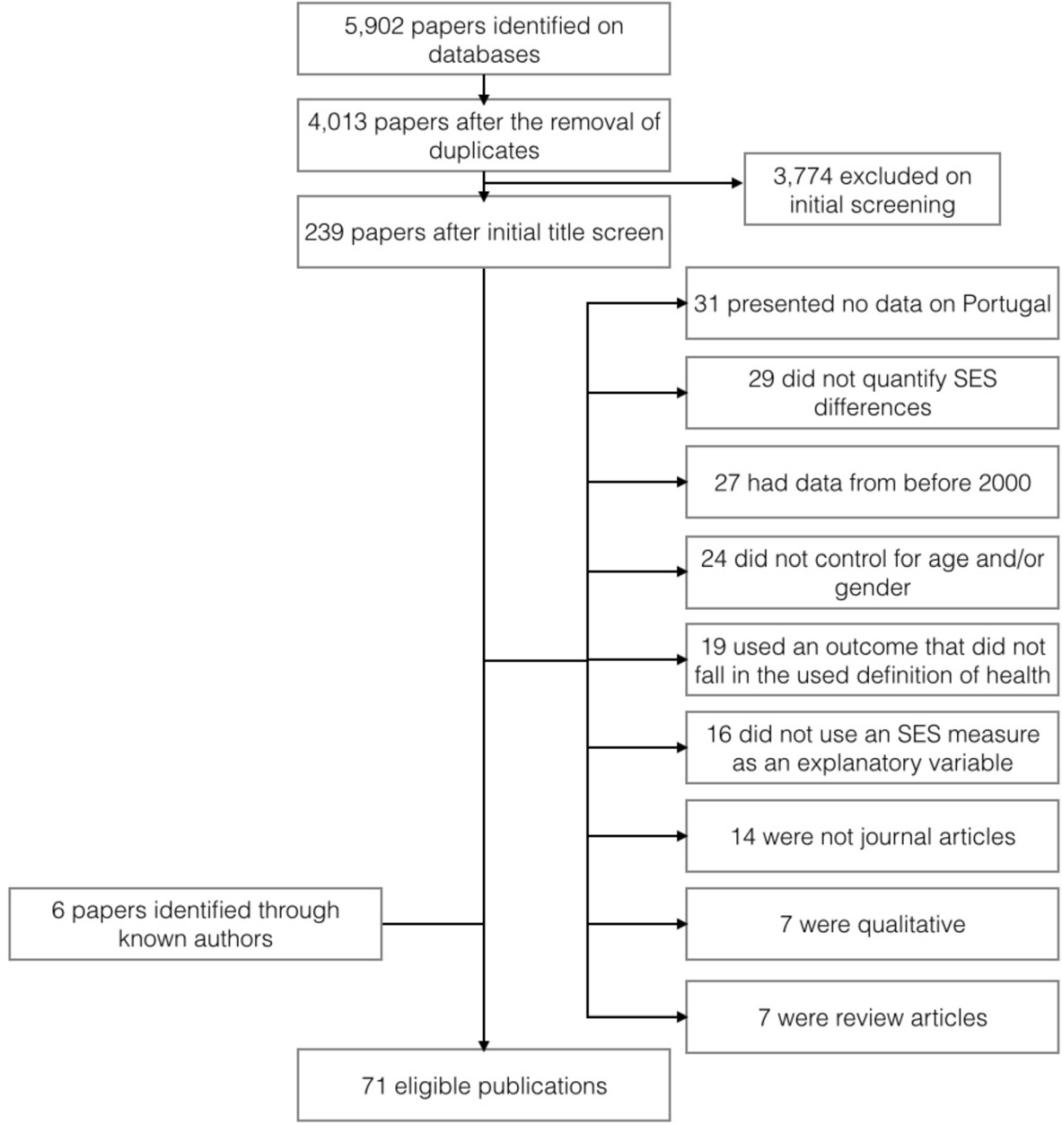
Process of study selection

Figure 1 shows the number of publications identified, screened, assessed for eligibility and included, with reasons for exclusion at each stage. Five thousand nine hundred and two publications were initially identified. After removing duplicates and the initial title screening, the most common reasons for exclusion were that no data for Portugal was presented (mostly international analyses that did not show country-specific results); SES differences were not quantified (the analysis did not compare SES groups); data was previous to the year 2000 or the analysis did not control for age and/or gender. In the end of this process, 71 publications were considered eligible. The complete extracted information from these 71 publications is in the online Additional file 2 and the list of references is in the online Additional file 4.

### Study characteristics

All eligible publications described observational data. The vast majority of these were cross-sectional and individual-level (five used ecological data). Five studies had a longitudinal design, three of which used data from the same cohort (the EpiPorto cohort, a cohort of community dwelling adults from Porto [12]). Other sources of data included the national health survey (a repeated cross-sectional nation-wide survey [13] (3 publications)), the Generation XXI cohort (a cohort of newborns and their mothers recruited in Porto between 2005 and 2006 [14] (3 publications)) and the EpiTeen cohort (a cohort of adolescents born in 1990 from Porto [15] (3 publications)). Sixteen publications used school-based samples, fifteen healthcare-based and twelve community-based.

Sample sizes ranged from 18 (municipalities in the Lisbon Metropolitan Area [16]) to over 800,000 (all national births over several years [17]), with an overall median of 1,234. The vast majority used regression analyses – linear and logistic – to quantify inequalities. Adults were the most commonly studied group (forty publications), followed by adolescents (twelve), children (seven) and newborns (four). Five analysed only women or girls and two had samples exclusively constituted my migrants.

Eligible studies focused on subjective health assessment, functional indicators and medical health. The majority (fifty-eight) used medical health outcomes, among which obesity and mental health were the most common, used in fifteen and fourteen publications, respectively. Subjective health assessments were used in twelve publications, mostly measured by self-assessed health. Finally, functional indicators were used in only ten publications, including measures of physical ability, cognitive ability, sickness absence and pain.

The most commonly used SES measure was education, used in thirty-three publications, followed closely by gender, used in twenty-eight. All other SES indicators were each used in less than twelve publications and were mostly measured at an individual level, except in the five ecological analyses and one multilevel analysis [18].

Table 1 summarizes the main results by health outcome and SES indicator. Publications looking at the association between place of residence showed that urban environment and deprivation were associated with worse health (see, for example, references [19] and [20]). For inequalities related to migration, mortality was consistently worse in migrants [21, 22] but migrant adolescents had better health [23–25]. Being employed or having a more differentiated occupation was either strongly or not associated with better health, never the opposite [21, 26–31]. Only two publications showed (some) worse health indicators for men as compared to women [32, 33]; otherwise, women consistently showed worse results for a variety of health outcomes [32, 34]. Only one study found an association between religion and life satisfaction [19]. Education was used in thirty-three publications, of which only three found an association between more education and worse health [19, 35, 36]; all others found strong associations between ill-health and lower education [20, 26, 31, 37–40]. Most publications that looked at health inequalities according to marital status found no association (see, for example, references [19, 31, 41]). Only six publications looked at income-related health inequalities and pro-poor inequalities were found in half of these [19, 28, 31]. Social capital was analysed using individual measures of social support and social activities, which were found to be associated with better health [28, 42].

**Table 1:** Description of main results of eligible publications, according to SES variable and health outcome used

		Health outcome		
		Medical indicators	Functional indicators	Subjective health
Socioeconomic determinants	Place of residence	Physical health tended to be better among rural adolescents (Machado-Rodrigues, 2012, Machado-Rodrigues, 2011) and less deprived neighborhoods (Bastos, 2013). Parental perceptions of better neighborhood environments also tended to show an association with better physical (Nogueira, 2013a, Machado-Rodrigues, 2014) but worse mental health (Carvalho, 2014) among children.	The only study (Nunes, 2010) showed no association between place of residence and cognitive ability.	One study (Humboldt, 2014) showed that life satisfaction was better in rural areas.
	Race/ethnicity/culture/language	Migrants showed higher mortality (Harding, 2008, Williamson, 2009), worse oral health (Pereira, 2013) and a higher percentage of small preterm births (Harding, 2006b). On the other hand, migrant adolescents had less mental health problems (Neto, 2009 and Neto, 2010) and better cardiorespiratory fitness (Santos, 2011).		There were differences in SRH among nationalities in one study (Dias, 2013), but all other studies showed no association between migration, ethnicity or nationality and subjective health (Malmusi, 2014 and Humboldt, 2014).
	Occupation	Most studies showed a strong association between unemployment or less differentiated occupations and worse health (see, for example, Fraga, 2014 or Santos, 2008), although some found no association (for example Alves, 2012 or Bastos, 2013). None found an opposite result.	One study (Azevedo, 2012) found people who were unemployed or retired were more likely to suffer from chronic pain.	Silva (2014) showed strong associations between employment and more differentiated occupations with SRH. On the other hand, Humboldt (2014) found no association between employment and life satisfaction.
	Gender/sex	Almost all studies showed an association between being female and worse health (see, for example, Santos, 2011 or Bulhões, 2013). Some studies found no gender differences (see, for example, Bastos, 2013 or Neto, 2010) and two found the opposite association (Perelman, 2012 and Stewart-Knox, 2012).	Women were more likely to take sickness absence (Masterkaasa, 2014 and Perelman, 2012) and report chronic pain (Azevedo, 2012 and Perelman, 2012), and one study showed men reported more bed days (Perelman, 2012). Cognitive abilities differed between genders, depending on the test used (Martins, 2012, Santos, 2014a).	Almost every study showed women had worse subjective health outcomes (see, for example, Bambra, 2009, Dias, 2013 or Pereira, 2011).
	Religion	One study showed no association between religion or spirituality and the onset of major depression (Leurent, 2013).		One study showed religious people showed higher life satisfaction (Humboldt, 2014), and another showed no association between religion or spirituality and quality of life or well-being (Vilhena, 2014).
	Education	Lower education tended to show a strong association with worse health in almost all studies (see, for example, Bastos, 2013 or Santos, 2010). There were two exceptions: Lawlor, 2005, who showed that insulin resistance was more common in children of more educated parents and Costa, 2008, who showed girls whose parents were more educated had more eating disorder symptomatology.	Education was strongly associated with cognitive ability (Martins, 2012, Nunes, 2010 and Santos, 2014a), chronic pain (Azevedo, 2012) and functional limitations (Eikemo, 2008, Knesebeck, 2006).	Better SRH was associated with higher education in all studies (see, for example, Knesebeck, 2006 or Silva, 2014) except one, that showed the opposite (Humboldt, 2014).
	Socioeconomic status	Married individuals tended to show better health outcomes (see, for example, Harding, 2008 or Williamson, 2009), but had higher odds of being obese (Alves, 2012 and Goulão, 2015). Income, deprivation and financial difficulties showed conflicting results: while most studies tended to show worse health outcomes for more deprived people (see, for example, Pereira, 2013 or Alves, 2012) or no association at all (see, for example, Correia, 2014 or Pimenta, 2011), there were some exceptions that showed, for example, lower prevalence of obesity among homeless people (Oliveira, 2012) or more insulin resistance among children with richer parents (Lawlor, 2005).	One study (Azevedo, 2012) found no association between marital status and chronic pain. Early life SES, as measured by height, was strongly associated with chronic pain in women (Perelman, 2014).	Objective income (Humboldt, 2014, Silva, 2014) and perceived income (Dias, 2013) were found to be associated with subjective health, but not marital status (Humboldt, 2014) or height, as a measure of early life SES (Perelman, 2014).
	Social capital	One study (Ferreira-Valente, 2014) showed that social support was associated with better psychological functioning.	One study (Ferreira-Valente, 2014) showed that social support had a strong association with physical functioning, but not pain intensity.	Number of activities outside the home was the only social capital indicator that showed an association with SRH (Silva, 2014). Other analyses showed no association (Vilhena, 2014, Silva, 2014).

*Note*: no eligible publication explored the relationship between ‘race/ethnicity/culture/language’ or ‘religion’ and functional indicators

*Legend*: SRH Self Rated Health. SES Socioeconomic Status

### Synthesis of results

**Fig. 2:**
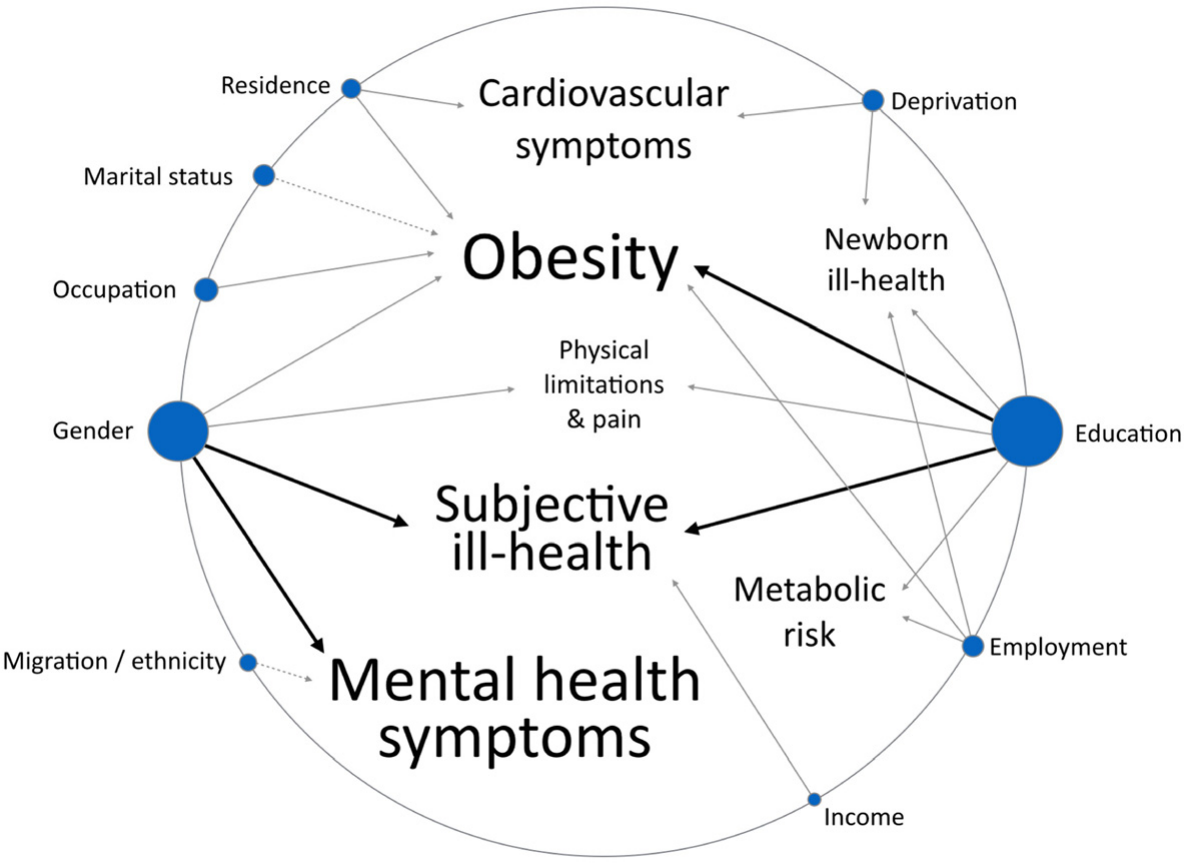
Diagram representing main results of the associations found in the eligible publications. The visual aspect of the diagram, but not the rules for its construction, was based on the diagram built by Ashley EA et al., “Clinical assessment incorporating a personal genome” The Lancet 375(2010): 1525-35. Note: Font size of health outcomes and circle size of socioeconomic determinants are proportional to the number of eligible publications in which they featured. Black arrows represent strong evidence of an association between socioeconomic indicator and health outcome; grey arrows represent weak evidence and dashed arrows represent evidence of the “negative” associations. In the results obtained, “negative” includes migrant populations having better mental health and married individuals having higher prevalence of obesity. Evidence of all other associations had a “positive” direction, i.e., ill health was associated with lower education, lower income, female gender, unemployment, deprivation, having less differentiated occupations and living in an unfavourable or urban area. Details on how this diagram was constructed are in the online Additional file 3

Figure 2 summarizes the associations found between SES indicators and health outcomes among the most commonly used variables. It stands out that obesity, mental health and subjective health were the most commonly employed health outcomes, and education and gender the most common SES variables. It is also clear that the strongest evidence for health inequalities exists between lower education and obesity and subjective ill-health, and between female gender and mental health symptoms and subjective ill-health.

Obesity and education were strongly linked in six publications, both for adults [31, 33, 43, 44] and for children and their parents’ education [45, 46]. The two of these studies that stratified the analyses by gender showed an effect only in women [43, 44]. One of these [43] had a longitudinal design and measured incidence rates of central and overall obesity in both women and men, showing a much lower rate in educated women (the adjusted risk ratio (RR) of women with over 11 years of education was 0.43 of that of those with less than 5 years, 95 % Confidence Interval (CI) = 0.22–0.84).

Education was also strongly linked to worse self-rated health in five publications, all using cross-sectional data. One showed no effect on men [39], but others showed an effect on both genders [47–49]. Schutte et al. (2013), for example, calculated that controlling for age, marital status and urbanization level, women in the lowest educational group were almost six times more likely to report poor health (Relative Index of Inequality (RII) = 5.9; 95 % Confidence Interval (CI) = 2.6–13.4) and men1.4 times (RII = 1.4; 95 % CI = 0.6–3.3) [39].

Strong evidence of gender health inequalities was also found. Of the six studies looking at gender and subjective health measures, only one - using a non-random sampling procedure - showed no association [19]. All others showed a strong association favouring men [28, 32, 47, 50, 51]. Perelman (2012), for example, used a community sample of over 30,000 randomly selected adults (from the National Health Survey) and after adjusting for other SES indicators such as income, marital status, occupation, employment, among others, showed that women still had between 1.4 and 2.3 higher odds of reporting bad SRH [32].

Female gender was also strongly linked to mental health symptoms. Of the nine studies looking at this link, two found no association, one of which used a non-random procedure and the other had no information on the sampling procedure [24, 52]. The other seven publications analysed children [53, 54], adolescents [23, 55] and adults [32, 34, 56], looking at a range of mental health outcomes, from depressive symptoms [55] to insomnia [53]. Santos (2014a), for example, used a random sample of adults over 50 registered in primary care from two health registries and showed that, after controlling for multiple medical conditions and health behaviours, women scored significantly higher on the Geriatric Depression Scale [56].

## Discussion

### Summary of evidence

This review identified the most studied health inequalities that have been evidenced in the literature for the Portuguese population since the year 2000. We selected 71 publications that explored a wide range of SES indicators and health outcomes, but strong evidence was found on health inequalities related to education and gender, mostly for obesity, SRH and mental health symptoms. In most cases, a large, significant and negative relationship was observed between SES and health outcomes.

Education was the most frequently studied determinant of health and for which most evidence exists of health inequalities. Evidence of educational inequalities in obesity was particularly common, especially for women, as the two studies that stratified the analysis by gender found only women showed significant inequalities [43, 44]. This suggests educational inequalities in overweight/obesity are found mostly or exclusively in women. This is not unique for Portugal: Roskam et al. (2010) found that other southern European countries also show high education inequalities in overweight and obesity only for women [57]. In this analysis, Portugal had the highest educational inequalities in overweight and obesity among women in all the countries analysed. This can be a consequence of various factors, such as inequalities in physical activity, dietary patterns or parity. However, both men and women seem to show the same extent of educational inequalities in physical activity and diet in Portugal [58, 59], which makes them unlikely factors in explaining inequalities in obesity seen mostly in women. On the other hand, women with lower education in Portugal have a higher fertility index [60], and since higher parity is strongly associated with obesity [61], this might be the most suitable explanation for the high educational inequalities in overweight and obesity seen for women in Portugal.

Education was also strongly associated with SRH [28, 39, 47–49], which is consistent with other international analyses [49, 62]. Interestingly a European comparison among 22 countries found that Portuguese men showed the highest education inequalities in SRH when compared to other countries [49]. However, educational inequalities in SRH should be interpreted with caution. As Huisman, Lenthe and Mackenbach (2007) pointed out, the predictive ability of SRH for mortality varies significantly among educational groups for men [63]. This probably reflects educational differences in men’s health perception, biasing the answers to questions on subjective health.

Our review also suggested strong gender inequalities in both SRH and mental health symptoms. Gender-related health inequalities is a broad and complex topic. Despite the prevailing notion that men have higher mortality and women higher morbidity [64], this has been challenged in the literature, and contradictory patterns continue to appear [65, 66]. Additionally, gender inequalities in health are probably a result of multiple factors, including biological and social [67], which raises questions of whether they should be considered as unfair or as unavoidable. Despite this, almost every publication that explored gender differences in our review showed strongly favourable results for men, particularly for mental health symptoms and SRH [32]. Noticeably, no publication explored gender differences in mortality.

Academic attention to health inequalities in Portugal has tended to focus on specific topics. Gender and education are by far the most commonly used SES indicators, possibly because they are the most easily measurable, commonly used in surveys with high response rates and high validity of answers and are less affected by reverse causation. Twelve publications also looked at health inequalities between migrants and Portuguese natives; this is surprising considering Portugal is one of the European countries with the lowest proportion of migrant population among its residents [68]. This could be imputed to both the ease of measurement of this variable and the presence of research groups in the country investigating this subject.

Other SES indicators appear to have been overlooked. For example, despite the growing literature on the effect of place in health, only a few publications explored this topic, most of which focused on rural/urban differences. There was also a notable deficiency of studies of social capital and poverty, despite Portugal’s high income inequality [6] and considerable risk of poverty and social exclusion [69]. Additionally, despite the growing recognition of the time dimension in the building of health inequalities [70], no publication took a life course approach to how SES indicators might affect health. This, coupled with the scarcity of longitudinal studies, substantially precludes the possibility of assessing causal relationships. This also speaks to a very scarce focus on the elderly - of the 71 eligible publications, only 7 focused on older people, which is surprising in a country where the old-age dependency ratio was the fifth highest in Europe in 2014 [71].

In 2013, the major causes of death in Portugal were diseases of the circulatory system (30), malignant tumours (24), diseases of the respiratory system (12), and endocrine, nutritional and metabolic diseases (5 %) [72]. In this sense, despite malignant tumours being the second most common cause of death, after circulatory diseases, there are strikingly few publications focusing on this health issue (four, of which two are ecological). This might again reflect the absence of a nationally oriented research policy, in part attributable to absence of political attention to this issue [3, 4, 8]. This is also the case for respiratory diseases, which are also almost absent from our analysis. In a recent report of a consortium published by the European Commission on Health Inequalities, Portugal was described as having “[clear] difficulties in measuring and analysing health inequalities” [73] (page 129). Interestingly, the current Portuguese National Health Plan identifies the reduction of child obesity as one of its four goals for 2020, but with no focus on its unequal distribution among socioeconomic groups [74]. This plan does mention the importance of the social determinants of health, but focuses almost exclusively on the access to health care services as a remedy for health inequalities [74].

The limited attention given to health inequalities in Portugal can only be explained with an extensive exploration of multiple factors, but one of these factors is probably the engrained belief that the National Health Service, as a universal and (relatively) inexpensive service at point of care, is enough to face these inequalities. However, this is apparently not true, as this review has shown there are still important health inequalities in Portugal. Tackling these inequalities will demand an important effort to build an organized research and policy strategy that will have to go beyond the National Health Service. It is important to notice that Portugal is amongst the most unequal countries in Europe, so that it could benefit from a more progressive taxation scheme and higher social protection to the poorest, which are major evidence-based and consensual measures to fight inequalities in health [75].

### Limitations

This review tried to bring together analyses not always comparable among them. In fact, many of these publications focused on specific populations – migrants, children or certain regions in Portugal – that might have particular patterns of health inequalities. This might have hidden inequalities that are not apparent when all groups are pooled together. Our search strategy might have also excluded important publications, namely international comparisons that included a Portuguese sample not specifically mentioned. However, we tried to overcome this by searching for publications by researchers known for having published in this area. The quality of the analyses in the reviewed publications was found to be heterogeneous, with some presenting highly reliable analyses and others relying on ‘convenience samples’, or on small sample sizes. Following the PRISMA guidelines, we chose not to score nor select the publications based on ‘quality’, but to carry out a brief assessment of strengths and limitations on each (table in Additional file 2). Also, we focused our review on papers published in indexed peer-reviewed journals according to good practices of scientific research, but this may have excluded important publications, in particular from the grey literature.

Finally, we restricted our analysis to health outcomes, and did not consider mediating factors such as lifestyle and healthcare use. Also, we did not consider studies on interventions to decrease inequalities in health. We adopted this strategy to avoid a too large scope for the review, which would have complicated the identification of general trends and interpretations. Further research should focus on these connected issues.

Along this paper, we referred to “inequalities” in health instead of other possible terms such as “inequity” or “differences”. In particular, inequity refers to differences that are unjust, unfair and avoidable [76]. This option was made because the concept of inequality is more neutral in terms of interpretations and value judgements, whereas the term “inequity” implies strong assumptions about the causes of differences, which none of the reviewed papers could confirm. Additionally, most reviewed papers referred to inequalities in health, so we opted to be faithful to authors’ interpretations.

## Conclusions

We have shown that there is strong evidence of socioeconomic health inequalities in Portugal and comparative analyses show that these are possibly one of the highest among European countries. We identified education and gender as the main determinants of health inequalities, affecting mostly the distribution of obesity, self-rated health and mental health symptoms. The publications we identified also reflect the absence of a nationally oriented research strategy on health inequalities focusing on the most prevalent diseases (such as malignant tumours and respiratory diseases), determining factors of inequalities (living contexts, poverty or social capital) and vulnerable populations (such as the elderly). We hope this review will help guide decision-making to tackle these issues, as has long been recommended.

## Additional files

Additional file 1:
**Detailed search strategy.** (PDF 79 kb) Additional file 2:
**Table of extracted data from the 71 eligible publications.** (PDF 196 kb) Additional file 3:
**Description of rules adopted to build diagram in Fig.** 2 **of the main text.** (DOCX 16 kb) Additional file 4:
**Complete list of the seventy one eligible publications identified by the systematic review, by alphabetical order.** (PDF 70 kb)
