# Supplementary material for: Connecting the dots on health inequalities – a systematic review on the social determinants of health in Portugal
Source: Int J Equity Health. 2016 Feb 16;15:26. doi: 10.1186/s12939-016-0314-z (PMC4754837; doi:10.1186/s12939-016-0314-z)
Supplement: Additional file 1: — Detailed search strategy. (PDF 79 kb) [file 12939_2016_314_MOESM1_ESM.pdf]

Additional File 1: Search Strategy

The PROGRESS framework was used as an initial framework to ensure the main determinants of socioeconomic health inequalities were covered in the search strategy.
From each term of this framework (first column of table 1), the closest MeSH terms were identified (second column of table 1 and corresponding entry terms in the third column). The MeSH terms in Table 1 were all included in the search as a Major Topic and connected by an “OR” Boolean operator. This search was performed with the term “Portug*”, connected by an “AND” operator.
To make sure articles not focusing exclusively on Portugal were included, a second search was performed by looking for the term “Portug*” in all fields and adding other terms referring to international comparisons. The following chart schematizes these two searches:

Search 1	Search 2
Table 1, (A)	Table 1, (A)
AND	AND
Portug*[MeSH]	Portug*[All Fields]
	AND
	(international[All Fields] AND comparison$[All Fields]) OR (european[All Fields] AND comparison$[All Fields]) OR (european[All Fields] AND countries[All Fields])

Both searches were restricted to articles published after January 1st 2000 in English or Portuguese.
Using the MeSH terms (and corresponding entry terms) as a starting point, the most appropriate terms for Web of Science and Scopus were then selected (fourth and fifth columns of table 1). This selection excluded terms that did not apply to the Portuguese context (such as “Minority Health and Health Disparities Research and Education Act of 2000”) and terms that were felt would amplify the search too much to little or no gain (such as “domicile”).
In both Web of Science and Scopus, these terms were searched for in the title, abstract or keywords of the publications. They were all included and connected by the “OR” operator. All databases in both aggregators were used and were limited to publications written in English or Portuguese published after January 1st 2000.
In Web of Science it is not possible to search for a term in all fields of a publication. Therefore, a search for publications on country comparisons retrieved an high amount of papers, many of which did not include Portugal. Thus, only two searches were performed in this aggregator, on articles focusing on Portugal:

Search 1	Search 2
Table 1, (B)	Table 1, (C)
AND	AND
Portug*	Portug*
AND	AND
health*	"saúde" OR "saud*"

Finally four searches were performed on Scopus:

Search 1	Search 2	Search 3	Search 4
Table 1, (B)	Table 1, (B)	Table 1, (C)	Table 1, (C)
AND	AND	AND	AND
Portug*	"international comparison$" OR "european comparison$" OR "european countries"	Portug*	"comparaç* internaciona*” OR "comparaç* europeia$" OR "países europeus"
AND	AND	AND	AND
health*	health*	"saúde" OR "saud*"	"saúde" OR "saud*"
	AND		AND
	Portug*		Portug*

PROGRESS Framework		MESH terms (A)	MESH Entry terms	Web of Science / Scopus (English) (B)	Web of Science / Scopus (Portuguese) (C)
P	Place of residence	Residence Characteristics	Domicile<br>Residential selection<br>Neighborhood<br>Place of birth / Birth place<br>Community<br>Living arrangements	"Poverty area$"<br>"Slum$"<br>"Ghetto$"<br>"Rural health"<br>"Urban health"<br>"Suburban health"<br>"Context*"<br>"Place of birth"<br>"Birthplace"	"Desigualdade$ geográfica$"<br>"Disparidade$ geográfica$"<br>"Gueto$"<br>"Saúde rural"<br>"Saúde urbana"<br>"Saúde suburbana"<br>"Context*"
		Poverty areas	Slum<br>Ghetto		
		Rural population	Rural Spatial Distribution Rural Community		
		Rural health			
		Urban population	Urban Spatial Distribution		
		Urban health			
		Suburban population	Nonmetropolitan Population<br>Suburbanization		
		Suburban health			
R	Race / ethnicity / culture / language	Continental Population Groups	Racial Stock<br>Race	"Race$"<br>"Ethnic group$"<br>"Nationalit*"<br>"Migrant$"<br>"Emigrant$"<br>"Immigrant$"<br>"Minority group$"<br>"Minority health"	"Raça$"<br>"Etnia$"<br>"Nacionalidade$"<br>"Migrante$"<br>"Emigrante$"<br>"Imigrante$"<br>"Minoria$"
		Ethnic groups	Nationality		
		Culture	Custom<br>Belief<br>Cultural background		
		Language			
		Transients and Migrants	Migrant Workers<br>Nonmigrant<br>Squatter<br>Illegal migrant<br>Nomad		
		Emigrants and Immigrants	Foreigner<br>Alien		
		Minority groups			
		Minority health	Minority Health and Health Disparities Research and Education Act of 2000		
O	Occupation	Occupations	Vocation	"Occupation$"<br>"Employment"<br>"Underemployment"<br>"Unemployment"	"Ocupaç*"<br>"Profiss*"<br>"Desemprego"<br>"Emprego"
		Employment	Occupational status<br>Employment termination<br>Informal sector<br>Underemployment<br>Employment status<br>Child labor<br>Labor force		
		Unemployment			
G	Gender / sex	Gender Identities	Sex role<br>Gender role<br>Man's role<br>Woman's role	"Gender inequalit*"<br>"Gender disparit*"<br>"Sex inequalit*"<br>"Sex disparit*"	"Desigualdade$ de género"<br>"Disparidade$ de género"
		Sex	Phenotypic Sex<br>Genotypic Sex		
R	Religion	Religion	Religious Beliefs<br>Religious Ethics<br>Prayer	"Religio* discrimination"	"Discriminação relig*"

E	Education	Education	Workshop<br>Training Programs<br>Educational Activities<br>Literacy Programs	"Education* disparit*"<br>"Education* inequalit*"<br>"Illiteracy"<br>“Schooling”<br>“Grad*”	“Disparidade$ educa*”<br>“Desigualdade$ educa*”<br>“Iliteracia”<br>“Escolaridade”<br>“Nota$”
		Educational Status	Educational Achievement<br>Illiteracy<br>Maternal Educational Status		
S	Socioeconomic status	Social Class	Socioeconomic Status<br>Middle Class Population<br>Caste	"Social Class"<br>"Socioeconomic Status"<br>"Socioeconomic Factor$"<br>"Standard$ of Living"<br>"Living Standard$"<br>"Inequalit*"<br>"Disparit*"<br>"Poverty"<br>"Income$"<br>"Remuneration"<br>"Salaries"<br>"Salary"<br>"Social mobility"<br>"Social determinant$ of health"<br>"Deprivation"<br>"Marital status"<br>“Affluen*”<br>“Consumption”<br>“Asset$”	“Classe$ socia*”<br>“Estatuto socio$económico”<br>“Fa$tor* socioeconómico$”<br>“Níve* de vida”<br>“Desigualdade*”<br>“Disparidade*”<br>“Pobreza”<br>“Rendimento$”<br>“Remuneraç*”<br>“Salário$”<br>“Mobilidade social”<br>“Determinantes sociais da saúde”<br>“Deprivação”<br>“Estado civil”<br>“Afluência”<br>“Afluente$”<br>“Consumo”<br>“Bens”
		Socioeconomic Factors	Standard of Living<br>Living Standard<br>Land Tenure<br>High-Income Population<br>Inequality		
		Health Status Disparities			
		Poverty	Indigent / Indigency<br>Low-Income Population		
		Income	Income Generation Programs<br>Savings<br>Income Distribution		
		Remuneration			
		Salaries and Fringe Benefits	Wage<br>Salary<br>Paternity Benefits<br>Pay Equity<br>Fringe Benefits		
		Social mobility			
		Social Hierarchy			
		Social determinants of health			
		Psychosocial deprivation			
		Social stigma			
		Marital status			
S	Social capital	Social capital		"Social capital"<br>"Social condition$"<br>"Living conditions"<br>"Social environment"<br>"Social support"<br>"Social network$"<br>"Social isolation"<br>“Social exclusion”	“Capital social”<br>“Condiç* social*”<br>“Condiç* de vida”<br>“Ambiente social”<br>“Apoio social”<br>“Rede$ socia*”<br>“Isolamento social”<br>“Exclusão social”
		Social conditions	Living conditions		
		Social environment	Social ecology		
		Community Networks	Community Care Networks<br>Community Health Networks		
		Social support	Social Network<br>Psychosocial Support Systems		
		Social isolation			
		Loneliness			
		Social alienation	Social Breakdown Syndrome		
		Social marginalization			
