# Supplementary material for: Connecting the dots on health inequalities – a systematic review on the social determinants of health in Portugal
Source: Int J Equity Health. 2016 Feb 16;15:26. doi: 10.1186/s12939-016-0314-z (PMC4754837; doi:10.1186/s12939-016-0314-z)
Supplement: Additional file 2: — Table of extracted data from the 71 eligible publications. (PDF 196 kb) [file 12939_2016_314_MOESM2_ESM.pdf]

Additional File 2: Extracted data from eligible publications

Reference	Population	Variables	Methods	Results	Strengths	Limitations
Alves et al, 2012	Consecutive sample of 7,381 puerperae from public maternities, Porto (Generation XXI)	Exposures: marital status, income, occupation, education and working conditions.<br>Outcomes: overweight/obesity, hypertension, dyslipidaemia and diabetes mellitus.<br>Control: age.	Logistic regression	Being obese/overweight was strongly associated with being married, lower education, less differentiated occupations, being unemployed or a "housewife" and having a lower income. Hypertension was less likely in highly educated women and more likely in "housewives" relative to "employed". Diabetes was inversely associated with income. Dyslipidaemia was not related to any SES indicator.	Probability sampling, large sample size.	Most outcome[illegible] were self-reported, cros[illegible] sectional data[illegible]
Amaral et al, 2013	School-based sample of 6,899 adolescents, aged 12-18, Viseu	Exposure: gender.<br>Outcome: insomnia.<br>Control: age.	Logistic regression	Female gender was associated with insomnia symptoms in adolescents (OR=1.82; 95% CI=1.56–2.13).	Large sample size.	Use of a “convenience sample”, cros[illegible] sectional data[illegible]
Azevedo et al, 2012	Random stratified sample of 5,094 adults, over 18	Exposures: gender, marital status, occupation and education.<br>Outcome: chronic pain.<br>Control: age.	Logistic regression	Women were more likely to have chronic pain than men (OR=2.37, 95% CI=2.03–2.77). Unemployed (OR=1.64, 95% CI=1.14–2.38) and retired people (OR=1.67, 95% CI=1.28–2.17) were more likely to have chronic pain when compared to full time employees. Low educational level was associated with increased probability of chronic pain. Marital status was not associated with chronic pain.	Large sample size, probability sampling.	Cross-section[illegible] data.
Bambra et al, 2009	No mention of sample size for Portugal, adults over 16 (EUROTHINE/NHS)	Exposure: gender.<br>Outcome: SRH.<br>Control: age.	Logistic regression	Women had higher odds of reporting bad or very bad SRH (OR= 2.01, 95% CI=1.87-2.15).	Probabilistic sampling procedure.	Cross-section[illegible] data.
Bastos et al, 2013	Random sample of 2067 adults, over 18, Porto (EPIPorto cohort)	Exposures: education, neighbourhood deprivation, occupation and gender.<br>Outcome: H. pylori infection.<br>Control: age and gender.	Poisson regression	Living in a deprived neighbourhood was associated with a higher prevalence of infection. The incidence rate of infection was lower among the more educated (≥10 vs ≤9: risk ratio = 0.25, 95%CI: 0.06–0.96). No evidence of gender or occupational differences.	Probability sampling, considerable sample size, longitudinal data.	Possibility of selection bias [illegible] follow-up.
Bettencourt et al, 2013	Sample of 600 consecutive hospital admissions due to acute heart failure, 6 month follow-up	Exposure: socioeconomic deprivation index (income, educational level and living alone).<br>Outcome: mortality.<br>Control: age, gender and admission brain natriuretic peptide.	Cox regression analysis	Deprivation was not strongly associated with mortality (the hazard ratio of all-cause death was 1.48, 95% CI=0.77–2.82).	Longitudinal data, adequate control for confounders.	Used an uncommon SE[illegible] measure, sma[illegible] sample size.
Bingham et al, 2013	Stratified random sample of 17,136 children, aged 3–10, mainland Portugal	Exposures: gender and parental education.<br>Outcome: overweight/obesity.<br>Control: age.	Logistic regression	Low paternal education was strongly related to the odds of being overweight or obese, while low maternal education was only related to the odds of being obese. Girls had higher odds of both obesity and overweight.	Trained technicians obtained anthropometric measures, probability sampling, large sample size.	Cross-section[illegible] data, low response rate (57%).

Borrell et al, 2014	207 small areas from the Lisbon metropolitan area.	Exposure: social deprivation (unemployment, manual workers, population aged 25–64 with primary education or lower, population aged 25–34 with a university degree and foreigners from low income countries).<br>Outcome: overall mortality.<br>Control: age.	Relative risks of smoothed standardize d mortality ratios.	In women, mortality was higher in neighbourhoods with higher deprivation index, proportion of unemployed people and proportion of adults with primary education. In men, this association was only seen for unemployment.	Objective health outcome.	Ecological desi[illegible] low number of deaths can lea[illegible] to low statistic[illegible] power.
Bulhões et al, 2013	School based sample of 1,988 13 year-olds, Porto	Exposures: gender and parental education.<br>Outcome: depressive symptoms.<br>Control: gender, parents' depression and education.	Logistic regression	The prevalence of depressive symptoms was 18.8% in girls and 7.6% in boys (p < 0.001). Parental education was not a determinant of depressive symptoms in either boys or girls.	Outcome was assessed with a validated instrument.	Cross-section[illegible] data.
Camões et al, 2010	Random sample of 1,621 adults, 18 and over, Porto (EPIPorto cohort)	Exposures: gender and education.<br>Outcomes: overall and central obesity.<br>Control: age, energy intake and leisure time physical activity.	Poisson regression	The incidence rate of central obesity was significantly higher in women (5.97, 95% CI: 5.09–7.03) when compared to men (2.38, 95% CI: 1.81–3.20). There was a significant inverse association between obesity and education in women.	Longitudinal design, probability sampling, outcomes assessed by trained researchers.	High loss to follow-up (66[illegible] were followed[illegible]
Campos-Matos et al, 2014	Sample of 712 health care workers from primary care centers	Exposure: occupation.<br>Outcome: overweight/obesity.<br>Control: age and gender.	ANCOVA	Health service personnel had more than 3 kg/m^2 higher BMI when compared to superior technicians. Nurses and doctors were not significantly different.	Outcome assessed by researchers.	Non-probabili[illegible] sampling, cro[illegible] sectional data[illegible] low response [illegible]
Carvalho et al, 2010	Sample of 442 adults, 18 and over	Exposure: gender.<br>Outcome: sexual desire.<br>Control: age and education.	MANCOVA	Women reported significantly lower levels of sexual desire when compared to men.	Use of validated instruments to measure health outcomes.	Non-probabili[illegible] sampling, cro[illegible] sectional data
Carvalho et al, 2014	Stratified school-based random sample of 17,911 adolescents, 10-17 years	Exposures: gender and perceptions of neighbourhood safety.<br>Outcome: emotional symptoms.<br>Control: school commitment, communication with family and school grades	Linear regression	Female gender (β=0.16, p=0.0001) and higher perception of a safe neighbourhood (β=0.03, p=0.024) were associated with more emotional symptoms.	Large sample size, probability sampling.	Not validated health measur[illegible] cross-section[illegible] data.
Correia et al, 2014	Consecutive sample of 7,472 puerperae from public maternities, over 18, Porto (Generation XXI)	Exposures: maternal education, occupation and income.<br>Outcome: impaired female fertility.<br>Control: previous pregnancy experience, age, pregnancy planning and behavioural characteristics	Logistic regression	Lower education was associated with higher infertility among primigravidae but not in multigravidae. Occupation and income were not related to infertility.	Probability sampling, large sample size.	Excludes wom[illegible] who could not pregnant, possibility of misclassificati[illegible]
Correia et al, 2015	Sample of 6,893 adult mothers of singletons, Porto (Generation XXI)	Exposures: grandparents' education and social class, maternal education and marital status.<br>Outcome: small for gestational age.<br>Control: maternal age and gravidity.	Logistic regression	Being small for gestational age was less likely in more educated (OR= 0.77, 95% CI=0.65–0.90) and in married women (OR=0.64, 95% CI = 0.47–0.86). No association was found between grandparents' education and social class and being small for gestational age.	Large sample size, high response rate.	Possibility of recall bias, cr[illegible] sectional data
Costa et al, 2008	Random sample of 2,036 13 year olds, Porto (EPITeen)	Exposure: parental education.<br>Outcome: eating disorders symptomatology.<br>Control: type of school, grade at school, age at menarche, BMI and depressive symptoms.	Linear regression	Parental education had a significant positive effect on girls' drive for thinness and body dissatisfaction scores and a significant negative effect in boys' bulimia and body dissatisfaction scores.	Probability sampling, extensive control for confounders.	Possibility of selection bias[illegible] (among schoo[illegible] and students).

Dias et al, 2013	Snowball sample of 1,375 adult immigrants, over 18, Lisbon	Exposures: gender, nationality, education and perceived income.<br>Outcome: SRH.<br>Control: age, reported chronic disease, experienced mental illness, physical exercise and concern about eating habits.	Logistic regression	Good SRH was reported by 66.7% of men and 56.6% of women ($p < 0.001$). Good SRH was associated with African and Brazilian origin (compared to Eastern European) and secondary/higher education. Among women, good health was also associated with perceived sufficient income.	Extensive control for confounders.	Cross-sectiona data.
Eikemo et al, 2008	Random sample of 3,410 adults, 18 and over (ESS)	Exposure: education.<br>Outcomes: SRH and limiting longstanding illness.<br>Control: age.	Rate differences	All rate differences, for women, men, in SRH or limitations, were statistically significant, such that people with less education had worse health.	Large sample size, probability sampling.	Cross-sectiona data.
Falcão et al, 2008	Random sample of 1,911 13-year-old urban adolescents (EPITeen).	Exposure: gender and maternal education.<br>Outcomes: asthma and rhinitis.<br>Control: age.	Chi-square test	Boys were more likely to have had a rhinitis diagnose (prevalence was 0.120 versus 0.092, p=0.014). There was no difference in asthma prevalence. There were no differences in asthma or rhinitis diagnosis regarding maternal education.	Probabilistic sampling procedure and objective measurement of outcome.	Possibility of selection bias (some schools refused to participate, so significant differences between stude who participat and students v missing data).
Ferrão et al, 2013	Sample of 2,690 children, aged 3-10, Porto	Exposure: parental perceptions of residential neighbourhood environments.<br>Outcome: obesity.<br>Control: age, gender, maternal education and school cluster.	Logistic regression	The odds of obesity were lower in neighbourhoods that were perceived as safe, pleasant and with well-maintained sidewalks.	Researchers took anthropometric measures, large sample size.	No information sampling procedures, cross-sectiona data.
Ferreira-Pinto et al, 2012	Aggregated statistics on 278 counties based on approx. 200,000 hospital admissions	Exposure: counties' economic development.<br>Outcome: mortality rates.<br>Control: age, gender and health care resources.	Linear regression	Counties with higher economical development had significantly higher mortality rates (coefficient = 1.696, p<0.001).	Objective health outcome, considerable sample size.	Ecological design, cross-sectional data
Ferreira-Valente et al, 2014	Sample of 324 patients with chronic musculoskeletal pain from health institutions, 18 or over	Exposure: social support.<br>Outcomes: pain intensity, physical functioning and psychological functioning.<br>Control: age and gender	Linear regression	Social support was associated with physical functioning and psychological functioning but not pain intensity.	Use of validated instruments to measure the exposure and outcome.	Non-probabilis sampling, sma sample size, cross-sectiona data.
Fraga et al, 2015	Random sample of 1205 adults aged 35-75, Porto (EPIPorto cohort)	Exposures: education and occupation.<br>Outcome: inflammatory markers.<br>Control: age, gender, marital status, current smoking, heavy drinking, inactivity, BMI, chronic disease and anti-inflammatory medication	Logistic regression	Both low education and undifferentiated occupation were associated with increased inflammatory markers.	Large sample size, objective outcome measure, extensive control for confounders, data collection by trained interviewers, probability sampling.	Self-reported health behavic cut-offs used f health outcom were depende on the distribu in the populati cross-sectiona data.

Gotsens et al, 2013	207 small areas from the Lisbon metropolitan area.	Exposure: social deprivation (unemployment, manual workers, population aged 25–64 with primary education or lower, population aged 25–34 with a university degree and foreigners from low income countries).<br>Outcome: injury mortality.<br>Control: age.	Relative risks of smoothed standardized mortality ratios.	There were higher mortality rates due to transport injuries, falls, homicides and all injuries in neighbourhoods with lower socioeconomic index for men. For women, suicide mortality was lower in neighbourhoods with higher social deprivation. There were no other important associations.	Objective health outcome.	Ecological design, low number of dea[illegible] can lead to lo[illegible] statistical pow[illegible]
Goulão et al, 2015	Spatial random sample of 1,736 migrants, Lisbon and Setúbal	Exposures: nationality, gender, time in Portugal and marital status.<br>Outcome: BMI.<br>Control: gender, age, education, marital status and birthplace.	Linear regression	Being married was associated with higher BMI, when compared to being single (β=0.55, p=0.019). Immigrants from São Tomé e Príncipe had higher BMI when compared to Brazilians (β=1.21, p=0.004). Living in Portugal for 10-14 years (β=1.15, p=0,004) or over 15 years (β=1.48, p<0,001) was associated with higher BMI when compared to less than 5. Gender was not associated with BMI.	High response rate (97,9%).	Weight and he[illegible] were self-reported, cros[illegible] sectional data
Harding et al, 2006a	All births in a year in a hospital, 4,227 newborns, Amadora-Sintra	Exposures: maternal migrant status, education and occupation.<br>Outcome: birth weight.<br>Control: maternal age, education, mode of delivery, smoking, parity, gestational age and child gender.	Linear regression	Among babies of Portuguese white mothers, manual occupations were associated with lower birth weight. Maternal education was not associated with birth weight in any group. There were no significant differences in birth weights between different ethnic groups.	Large sample size, controlled for most important possible confounders.	Use of hospita[illegible] records, with considerable missing information.
Harding et al, 2006b	All births registered in Portugal (1995 – 2002), 872,058 newborns	Exposure: migration status.<br>Outcome: birth weight.<br>Control: year of birth, gender, maternal age, gestational age, and parity	Polytomous logistic regression	There was no difference in overall mean birth weights between Portuguese and African babies, but the percentage of small preterm births was higher among African (4.7%) than among Portuguese (2.9%) births.	Large sample size, analyses the whole population, not a sample.	Exposure is nationality, not migration statu[illegible]
Harding et al, 2008	Data from death registrations, 1998–2002, over 15,000 deaths.	Exposures: migration, marital status and occupational class (for men).<br>Outcome: cardiovascular mortality.<br>Control: age.	Death rates	African migrants had higher mortality for all causes, circulatory disease, coronary heart disease and stroke. There was considerable heterogeneity among Africans with Cape Verdeans having higher mortality than Angolans or Mozambicans. Occupation was associated with heart disease mortality rate for African but not for Portuguese men. Married individuals had lower mortality.	Analysis of all deaths in the time period, large sample size.	Change in ICD[illegible] codes in the middle of the period analyse[illegible]
Hoffman et al, 2014	207 small areas from the Lisbon metropolitan area.	Exposure: social deprivation (unemployment, manual workers, population aged 25–64 with primary education or lower, population aged 25–34 with a university degree and foreigners from low income countries).<br>Outcome: avoidable mortality.<br>Control: age.	Relative risks of smoothed standardized mortality ratios.	Deaths due to AIDS, cervical or uterine cancer, cerebro-vascular diseases and congenital heart diseases were higher in more deprived neighbourhoods. Mortality due to malignant colon illness was higher in less deprived neighbourhoods. There was no association between social deprivation and malignant diseases of the rectum, anal area or testes, or Hodgkin's disease, rheumatic heart disease, hypertension, heart failure, peptic ulcer, renal failure or conditions from the perinatal period.	Objective health outcome.	Ecological design, low number of dea[illegible] can lead to lo[illegible] statistical pow[illegible]

Humboldt et al, 2014	Sample of 1,234 adults from life-long learning centers, over 75, Lisbon and the Algarve	Exposures: gender, education, marital and professional status, income, urban-rural residence, religion and nationality.<br>Outcome: life satisfaction.<br>Control: age, recent disease, physical activity, medication.	Structural equation modelling	Having a religion (β=0.725; p<0.001), higher income (β=0.551; p<0.001), lower education (β=−0.403; p<0.001) and living in a rural area (β=−0.292; p<0.001) were associated with higher life satisfaction. Other SES variables were not significantly related to life satisfaction.	Adequate control for confounders.	Non-probabilis sampling, cros sectional data
Knesebeck et al, 2006	Random sample of 1,312 adults, 25 or over (ESS)	Exposure: education.<br>Outcomes: SRH and functional limitations.<br>Control: age.	Logistic regression	Lower education was strongly associated with worse SRH and more functional limitations, both in women and in men.	Probability sampling.	Cross-sectiona data.
Lawlor et al, 2005	School-based random sample of 1,153 children, aged 9 and 15, Madeira	Exposures: family income and parental education.<br>Outcome: insulin resistance.<br>Control: age, sex, parental BMI, birth weight, breast-feeding, height, pubertal stage, BMI and waist circumference.	Linear regression	Lower income and lower parental education were associated with lower insulin resistance.	Probability sampling, extensive control for confounders.	Income was no equivalised fo family size, cro sectional data
Leurent et al, 2013	Consecutive sample of 1,005 adults, aged 18–75, from primary care, 6-12 months follow-up, Lisbon	Exposures: spiritual and religious beliefs.<br>Outcome: major depression.<br>Control: age, sex, education, employment, social support and past history of depression.	Logistic regression	There were no significant differences in onset of major depression between religious (OR=1.78, 95% CI=0.39–8.08) or spiritual people (OR=1.52, 95% CI=0.27–8.48) when compared to neither religious nor spiritual.	Longitudinal data.	Unclear sampl methods and response rate
Machado-Rodrigues et al, 2011	Sample of 362 adolescents, 13–16 years of age, midlands (MALS)	Exposures: urban-rural residence and parental education.<br>Outcome: CRF.<br>Control: age, weight status and physical activity.	Logistic regression	Adolescents of both sexes from rural settings were 76% more likely to be classified as aerobically fit compared to those from urban areas. Higher maternal education was also a predictor of better CRF in girls.	Researchers assessed CRF, adequate control for confounders.	Cross-sectiona data, small sample size.
Machado-Rodrigues et al, 2012	School-based stratified random sample of 362 adolescents, aged 13-16, midlands	Exposure: urban-rural residence.<br>Outcomes: CRF and BMI.<br>Control: Age	ANCOVA	CRF was better in both rural boys and girls. There were no statistically significant differences in BMI according to place of residence.	Researchers objectively assessed CRF and BMI.	Cross-sectiona data, small sample size.
Machado-Rodrigues et al, 2014	Stratified random sample of 1,886 girls aged 7–9 years.	Exposure: parental perceptions of neighbourhood environments.<br>Outcome: obesity and overweight.<br>Control: age, time spent in organized sports and parental education.	Linear regression	Neighbourhoods with interesting things to look at while walking were significantly associated with higher BMI (Beta =0.057, p=0.02), and neighbourhoods with many stores within easy walking distance were significantly associated with lower BMI (beta=-0.065, p=0.01).	Weight and height were collected by trained researchers.	Cross-sectiona data.
Malmusi, 2014	Stratified random sample of approximately 12,000 adults (EU-SILC)	Exposure: migrant status.<br>Outcome: SRH.<br>Control: age.	Poisson regression	There were no differences in age-adjusted prevalence of poor health between migrants and natives, neither in women nor in men.	Large sample size, probability sampling.	Cross-sectiona data, little con for other possi confounders.

Marí-Dell'Olmo et al, 2015	207 small areas from the Lisbon metropolitan area.	Exposure: social deprivation (unemployment, manual workers, population aged 25–64 with primary education or lower, population aged 25–34 with a university degree and foreigners from low income countries).<br>Outcome: mortality.<br>Control: age.	Relative risks of smoothed standardized mortality ratios.	In men, higher mortality in more deprived areas was found for respiratory diseases, chronic liver diseases, cerebrovascular diseases, influenza and pneumonia and diabetes. In women, the same relationship was found for ischemic heart disease, chronic liver disease, cerebrovascular disease and diabetes. An opposite association was found for lung cancer and breast cancer in women. No associations were found for ischemic heart disease, lung cancer or prostatic cancer in men and for respiratory diseases and influenza and pneumonia in women.	Objective health outcome.	Ecological desi[illegible] low number of deaths can lea[illegible] to low statistica[illegible] power.
Martins et al, 2012	Sample of 479 adults attending primary care in two metropolitan areas, over 50	Exposures: gender and education.<br>Outcome: executive function.<br>Control: age.	Linear regression	Lower educational levels were significantly associated with worse executive function. Gender was associated with some, but not all tests, and the direction of this association depended on the test.	Assessment of outcome by trained researchers.	Possibility of selection bias, cross-sectiona[illegible] design.
Mastekaasa, 2014	Random sample of adults, aged 20-59 (EULFS)	Exposure: gender.<br>Outcome: sickness absence.<br>Control: age, living with partner, children, level of education, working hours, occupation and industry.	Poisson regression	There were statistically significant gender gaps in sickness absence in Portugal, with OR that ranged from 1.27 to 2.22 in all the years analysed (women had higher odds).	Very high response rate (91%) for Portugal.	No information sample size fo[illegible] Portugal, cros[illegible] sectional data
Mello et al, 2008	School-based sample of 700 13 year olds, Porto.	Exposures: type of school, maternal education and gender.<br>Outcome: dental caries.<br>Control: soft drinks consumption.	Logistic regression	Attending a public school, being female and having parents with low educational attainment were identified as risk factors both for having dental caries and for having a high level of dental caries.	Caries registered by one trained dentist.	Cross-sectiona[illegible] data, non-probabilistic sampling.
Miranda et al, 2014	18 municipalities, Lisbon metropolitan area	Exposures: illiteracy rate, deprivation, unemployment rate and proportion of precarious households.<br>Outcome: pre-term births.<br>Control: maternal age.	Relative risk & Moran's I	There was a global significant association between the relative risk of preterm births and illiteracy rate (Moran's I=0.44), deprivation (Moran's I=0.32) and the unemployment rate (Moran's I=0.26). There was no association with precarious households.	Adequate methods.	Ecological design, cross-sectional data
Neto, 2009	Sample of 1,055 adolescents (partially from ICSEY), Lisbon	Exposures: migration status and gender.<br>Outcome: mental health problems.<br>Control: age and SES.	ANCOVA	Adolescents from immigrant families reported fewer mental health problems than their native Portuguese counterparts, and girls reported more mental health problems than boys.	Large array of instruments to measure mental health problems.	Cross-sectiona[illegible] data, non-probabilistic sampling.
Neto, 2010	Sample of 322 adolescents, aged 13-19, north of Portugal	Exposures: migration status and gender.<br>Outcomes: depression, anxiety and psychosomatic symptoms.<br>Control: age.	ANCOVA	Adolescents from immigrant families reported fewer mental health problems than Portuguese adolescents who have never migrated. There were no gender differences.	Use of a control group.	Non-probabilis[illegible] sampling, sma[illegible] sample size, cross-sectiona[illegible] data.
Nogueira et al, 2013a	School-based sample of 1,885 children, 3–10 years, Coimbra	Exposure: parents' perceptions of social and built residential environment.<br>Outcome: obesity.<br>Control: age and parental education.	Logistic regression	Girls living in neighbourhoods perceived as having poorly built environmental conditions and as being unsafe had increased odds of being obese (OR=1.47 and 1.34, respectively, p<0.005). These relationships were not evident for boys.	Researchers measured weight and height.	Recruitment a[illegible] sampling procedures ar[illegible] not described, cross-sectiona[illegible] data.

Nogueira et al, 2013b	Sample of 1,885 Portuguese children, aged 3–10, Coimbra	Exposure: parental education.<br>Outcome: obesity.<br>Control: gender, age and clustering of children in schools.	Logistic regression	Children whose parents had low (OR = 51.76, 95% CI=1.25–1.99) and medium (OR=1.57, 95% CI=1.34–2.33) education were more likely to be obese than their high-education peers.	Large sample size.	No information selection or sampling procedures, cross-sectiona data.
Nunes et al, 2010	Random sample of approximately 1,000 adults, aged 55-79, primary care registries, Northen Portugal	Exposures: urban-rural residence and education.<br>Outcomes: cognitive impairment and dementia.<br>Control: age, gender, vascular risk factors, cardiovascular disease, depression and other diseases.	Logistic regression	Lower education was associated with cognitive impairment (OR = 1.54, 95% CI=1.02-2.33), whereas residence was not. There were no significant associations with dementia.	Probabilistic sampling.	Cross-sectiona data, high non response rate 48%).
Oliveira et al, 2012	Sample of 146 homeless adults, over 18, and matched controls (on sex, age and education) from the general population, Porto	Exposure: homelessness.<br>Outcomes: overweight/obesity, abdominal obesity, hypertension, dyslipidaemia and diabetes.<br>Control: age and education.	Poisson and linear regression	Overweight/obesity (prevalence ratio=0.66, 95% CI=0.45-0.95) and self-reported dyslipidaemia (prevalence ratio=0.21, 95% CI=0.10-0.43) were less common among homeless participants than in non-homeless. There were no differences in the other health outcomes.	Some anthropometric measures taken by researchers, use of matched control group.	Most outcome are self-report cross-sectiona data.
Oliveira et al, 2015	Sample of 96,905 hospital patients with hip fracture, 50 and over, within 278 municipalities of continental Portugal	Exposure: municipal deprivation.<br>Outcome: hip fracture.<br>Control: age and gender.	Hierarchical regression model	In women, there was a lower risk associated with more affluent municipalities: relative risk=0.83 (95%CrI 0.65–1.00). In older ages (≥75 years) affluent municipalities had higher risk of hip fracture.	Large sample size, use of multilevel data and methods.	Cross-sectiona data.
Pereira et al, 2011	Sample of 1,191 HIV-positive adults, over 18.	Exposure: gender.<br>Outcome: quality of life.<br>Control: time since diagnosis, CD4 counts and HIV stage.	MANCOVA and linear regression	There was a significant effect of gender on quality of life, as women tended to report lower scores.	Adequate control for potential confounders.	Non-probabilis sampling, cros sectional data
Pereira et al, 2013	Sample of 146 homeless adults, over 18, Porto	Exposures: gender, education, nationality and duration of homelessness.<br>Outcome: oral caries.<br>Control: age.	Linear regression	Having decayed teeth was significantly associated with nationality ('other' vs. 'Portuguese', β = 2.7, 95% CI=0.4-5.2) and years of homelessness ('>=6' vs '<=1 month', β=2.8, 95% CI=0.4,5.2) but not associated with gender or education. Having missing teeth was not associated with any of these variables.	A single dentist assessed outcome.	Cross-sectiona data, not a probabilistic selection procedure.
Perelman et al, 2012	Random sample of 33,662 adults, over 18 (NHS)	Exposure: gender.<br>Outcomes: SRH, restricted-activity days, bed days and chronic diseases.<br>Control: age, education, employment status, income, insurance status, marital status, occupation.	Logistic regression	Women were more likely to have poor SRH, more days lost to disability, and 6 out of 8 chronic diseases. Men experienced more bed days.	Probability sampling, large and representative sample.	Cross-sectiona data and self-reported information on height and hea conditions.

Perelman, 2014	Random sample of 28,433 adults, aged 25-79 (NHS)	Exposure: height (as an indicator of early-life SES). Outcomes: SRH and chronic diseases. Control: age, gender, obesity, smoking, employment and education.	Logistic regression	Height was associated with the risk of several chronic diseases (asthma, chronic pain, cardiovascular disease, mental disease), but this association is largely mediated by education among men.	Probability sampling, large and representative sample.	Cross-sectiona data and self-reported information on height and hea conditions.
Pimenta et al, 2011	Sample of 243 women with vasomotor symptoms, aged 42-60, Lisbon	Exposures: marital status, professional status, income and education. Outcomes: Hot flashes and night sweats. Control: perceived control, age, parity, menopause, health care use, therapy for menopausal symptoms, psychological problems, alcohol and coffee intake, smoking, physical exercise and BMI.	Structural equation modelling	No SES variable showed any association with the health outcomes.	Extensive control for potential confounders.	Recruitment ar sampling procedures ar not described, cross-sectiona data, small sample size.
Ramos et al, 2007	Random sample of 2,161 13-year-old urban adolescents (EPITeen).	Exposure: parental education. Outcome: overweight. Control: parental smoking and BMI, gender, family structure, school characteristics, birth weight, breastfeeding practice, age at menarche, sleep duration and leisure time activities.	Logistic regression	There was no statistical association between overweight children and parental education.	Weight and height were collected by trained researchers.	Cross-sectiona data.
Ribeiro et al, 2014	Sample of 97 centenarians, Porto and Beira Interior	Exposure: gender. Outcome: anxiety symptoms.	Logistic regression	There was no difference in anxiety symptoms between men and women (OR=4.29, 95%CI=0.88–21.05)	Use of validated instruments to measure outcome.	No information selection or sampling procedures, cross-sectiona small sample s
Rodrigues et al, 2008	Sample of 1,822 consecutive births from public maternities.	Exposure: maternal employment. Outcome: pre-term delivery. Control: maternal age, marital status, education and obstetric characteristics.	Logistic regression	Women entering pregnancy while unemployed presented a significantly increased risk of spontaneous preterm delivery (OR=1.5, 95% CI=1.18–1.88).	Use of control group, controlled for most important confounders.	Possibility of b due to health selection, non-probabilistic sampling.
Ruiz et al, 2015	Consecutive sample of 8,330 births from public maternities, Porto (Generation XXI)	Exposure: maternal education. Outcomes: pre-term birth and small for gestational age. Control: child sex, maternal age and ethnicity.	Relative index of inequality and slope index of inequality	There was no association between maternal education and pre-term birth. There was a significant association between maternal education and being small for gestational age (RII=1.29, 95% CI = 1.01;1.58 and SII=2.90, 95% CI = 0.20;5.60).	Probability sampling and large sample size.	Missing data v more common among mother with low education – possibility of b
Santana et al, 2014	All diabetes deaths per municipality in Portugal, covering 278 municipalities.	Exposure: index of sociomaterial deprivation (illiteracy, unemployment and housing without toilets). Outcome: diabetes mortality. Control: age and gender.	Bayesian hierarchical model	After the year 2000, the relative risk of death by Diabetes Mellitus according to vulnerability associated to social and economic conditions in the area of residence was not significant (relative risk: 1.00; IC95%: 0.98-1.02).	Observes all population (all deaths), objective outcome.	Ecological design, cross-sectional data
Santos et al, 2003	Random sample of 1,436 adults, aged 18–90, Porto	Exposures: education, occupation and marital status. Outcome: obesity. Control: age, smoking status, physical activity and energy intake	Logistic regression	The prevalence of obesity was significantly higher in women (26.1%) than in men (13.9%). In women, the odds of obesity were higher in the less educated. In men, there was no association between obesity and any of the SES variables.	Outcome was measured by researchers, probability sampling.	Cross-sectiona data.

Santos et al, 2008	Random sample of 1,962 adults over 40 years, Porto.	Exposures: marital status, education, occupation and social class. Outcome: metabolic syndrome. Control: age, BMI, blood pressure, physical activity, alcohol consumption and smoking.	Logistic regression	Among women, lower education, more differentiated occupation and lower social class, but not marital status, were associated with higher odds of metabolic syndrome. There were no significant associations among men.	Anthropometric measures were taken by trained researchers, probability sampling and large sample size.	Cross-section[illegible] data.
Santos et al, 2010	Random sample of 1,093 adults, over 18, Porto (EPIPorto cohort).	Exposures: gender and education. Outcome: metabolic syndrome. Control: age, blood pressure, waist circumference, cholesterol, glucose and triglycerides.	Poisson regression	Low education was associated with 1.53 higher odds of developing metabolic syndrome ($p<0.05$). There were no gender differences.	Longitudinal data, extensive control for confounders, probability sampling.	High loss to follow-up (23[illegible]
Santos et al, 2011	School based sample of 266 adolescents, aged 12-18, Lisbon.	Exposures: gender and ethnicity. Outcome: CRF. Control: age and percentage body fat.	Linear regression	Interactions between age and ethnicity and between age and gender showed negative associations with CRF, such that Caucasian adolescents and girls had lower cardiorespiratory fitness.	Researchers objectively assessed CRF.	Non-probabili[illegible] sampling, cros[illegible] sectional data[illegible] small sample [illegible]
Santos et al, 2014a	Random sample of 1,051 adults, 50 and over, from health registries, Guimarães and Vizela.	Exposures: education and gender. Outcomes: cognitive abilities and mood. Control: age, physical activity, alcohol consumption, chronic diseases and BMI.	Linear regression and structural equation modelling	Gender showed different associations with cognitive ability, depending on the test used. Women tended to show more depressive mood. Education was positively associated with cognitive ability.	Probability sampling, measures are confirmed by medical records.	Cross-section[illegible] data.
Santos et al, 2014b	School-based sample of 517 adolescents, aged 15–18, Azores	Exposure: parental education. Outcomes: body fat, systolic blood pressure, triglycerides, cholesterol, insulin resistance and metabolic risk score. Control: age and gender.	Z-scores	Systolic blood pressure and metabolic risk score were higher in adolescents whose parents had lower education. The other outcomes were not associated with parental education.	Outcomes were objectively tested.	Cross-section[illegible] data.
Schutte et al, 2013	Stratified random sample of approximately 1,000 adults (EQLS)	Exposure: education. Outcome: SRH. Control: age, marital status and urbanization.	RII	Only women showed significant education-related inequality in SRH (RII for men = 1.4 (0.3, 3.3) and women = 5.9 (2.6, 13.4)).	Adequate methods.	Cross-section[illegible] data.
Silva, 2014	Randomized stratified sample of 1,000 adults over 50, continental Portugal	Exposures: gender, occupation, employment, income, education, individual social capital indicators (characterization of social network, characterization of social activities). Outcome: SRH. Control: age.	Linear regression	Being male, with more education, more differentiated occupation, employed, with higher income and higher number of activities outside the home were all associated with better SRH. Other social capital indicators had no association with SRH.	Probability sampling, extensive control for potential confounders.	SRH was analysed as a continuous measure, cros[illegible] sectional data[illegible]
Sousa-Ribeiro et al, 2014	Sample of 300 adults aged between 40 and 65, Porto.	Exposure: employment. Outcome: psychological well-being. Control: gender, parental status, civil status and education.	MANOVA	The employed reported better well-being than the other groups, and the unemployed in training showed lower distress than those who were not.	Used validated instruments to measure the health outcome.	Use of a "convenience sample".
Stewart-Knox et al, 2012	Stratified cluster sample of 540 adults, aged 43-93	Exposures: employment, gender and education. Outcomes: waist circumference and BMI. Control: dietary habits, physical activity, resilience, mood, hopelessness, perceived stress and life events.	Linear regression	BMI was not predicted by any SES variable. Being male, not working and having lower education were associated with higher waist circumference.	Researchers took anthropometric measures, probability sampling.	Cross section[illegible] data.

Vilhena et al, 2014	Sample of 774 chronic disease patients, over 17, from hospitals	Exposures: spirituality and social support.<br>Outcomes: quality of life and subjective well-being.<br>Control: gender, education, age, time since diagnosis and severity of disease perception	MANCOVA	Spirituality and social support were significant predictors of quality of life and subjective well-being.	Used validated instruments to measure health outcome, extensive control for potential confounders.	Unclear sampl procedures, cross-sectiona data.
Williamson et al, 2009	All death registrations, 1998–2002, over 5,000 deaths.	Exposures: migration status, gender, marital status and occupational class (for men).<br>Outcome: infectious disease mortality.<br>Control: age.	Death rates	Compared with people born in Portugal, African migrants had higher mortality for infectious diseases including AIDS. Death rates were higher among unmarried people and men from manual occupational classes.	Objective health outcome, analysis of all deaths in the time period.	Change in ICD codes in the middle of the period.

Legend:

AMI Acute Myocardial Infarction. ANCOVA Analysis of Covariance. CI Confidence Interval. COPD Chronic Obstructive Pulmonary Disease. CRF Cardiorespiratory Fitness.BMI Body Mass Index. CrI Credible Interval. EPITeen Epidemiological Health Investigation of Teenagers in Porto.EQLS European Quality of Life Survey. ESS European Social Survey.EU-SILC European Union Survey on Income and Living Conditions. EULFS European Union Labour Force Survey. EUROTHINE Health Inequalities in Europe. ICD International Classification of Diseases. ICSEY International Comparative Study of Ethnocultural Youth. MALS Midlands Adolescent Lifestyle Study.MANCOVA Multivariate Analysis of Covariance. NHS National Health Survey. RII Relative Index of Inequality. SES Socioeconomic Status. SRH Self Rated Health.
