# Supplementary material for: Connecting the dots on health inequalities – a systematic review on the social determinants of health in Portugal
Source: Int J Equity Health. 2016 Feb 16;15:26. doi: 10.1186/s12939-016-0314-z (PMC4754837; doi:10.1186/s12939-016-0314-z)
Supplement: Additional file 3: — Description of rules adopted to build diagram in Fig. 2 of the main text. (DOCX 16 kb) [file 12939_2016_314_MOESM3_ESM.docx]

**Additional file 3: Rules for building diagram in figure 2.**

The diagram presented on figure 2 was built according to the following rules:

- Health outcomes are presented inside the circle. Only health outcomes that were used in six or more eligible publications are used. The font size is proportional to the number of publications in which that outcome is used.
- SES variables are represented by circles in the circumference perimeter. Only SES variables that were used in six or more eligible publications are presented. The size of the circles is proportional to the number of publications in which the SES variable is used.
- Main findings from eligible publications were broken down to identify every combination of SES variable – health outcome analyzed. Each association was categorized as “positive”, “negative” or “null”.
  - Associations were considered “positive” if ill-health was associated with lower education, lower income, female gender, unemployment, deprivation, being a migrant or belonging to an ethnic group other than Caucasian, not being married, having a less differentiated occupation and living in an unfavorable or urban area.
  - Associations were considered “negative” if there was a significant association in the opposite direction.
  - Associations were considered “null” if they were not significant.
- Arrows in the diagram represent these associations. These were constructed based on the following rules:
  - *Grey* arrows indicate *weak* evidence. Evidence was considered weak when the difference between the number of “positive” and “negative” associations found was two or three. For example, obesity was found to be associated with female gender in three analyses and with male gender in one; this was considered to be weak evidence that female gender was associated with obesity.
  - *Black* arrows indicate *strong* evidence. Evidence was considered strong when the difference between the number of “positive” and “negative” associations was more than three. For example, worse subjective health was associated with lower levels of education in five analyses, and the opposite in one. This was considered strong evidence that higher education is associated with better self-rated health.
  - When there was only a difference of one analysis on a particular pair of SES variable and health outcome, it was considered insufficient evidence and excluded from the diagram.
  - When there were equal number of “positive” and “negative” associations, the result was considered contradictory and was not included in the diagram.
  - Non-significant results and associations in which “null” results were as common as either “positive” or “negative” were also excluded from the diagram.
- The visual aspect of the diagram, but not the rules for its construction, was based on the diagram built by Ashley EA et al., “Clinical assessment incorporating a personal genome” *The Lancet* 375(2010): 1525-35.
