# Supplementary material for: Connecting the dots on health inequalities – a systematic review on the social determinants of health in Portugal
Source: Int J Equity Health. 2016 Feb 16;15:26. doi: 10.1186/s12939-016-0314-z (PMC4754837; doi:10.1186/s12939-016-0314-z)
Supplement: Additional file 4: — Complete list of the seventy one eligible publications identified by the systematic review, by alphabetical order. (PDF 70 kb) [file 12939_2016_314_MOESM4_ESM.pdf]

Additional file 4: References of eligible publications.

Alves E, Correia S, Barros H, Azevedo A. Prevalence of self-reported cardiovascular risk factors in Portuguese women: a survey after delivery. International journal of public health. 2012;57(5):837-47.

Amaral MOP, Pereira CMF, Martins DIS, Sakellarides CT. Prevalence and risk factors for insomnia among Portuguese adolescents. European journal of pediatrics. 2013;172(10):1305-11.

Azevedo LF, Costa-Pereira A, Mendonça L, Dias CC, Castro-Lopes JM. Epidemiology of chronic pain: a population-based nationwide study on its prevalence, characteristics and associated disability in Portugal. The journal of pain. 2012;13(8):773-83.

Bambra C, Pope D, Swami V, Stanistreet D, Roskam A, Kunst A, et al. Gender, health inequalities and welfare state regimes: a cross-national study of 13 European countries. Journal of Epidemiology and Community Health. 2009;63(1):38-44.

Bastos J, Peleteiro B, Barros R, Alves L, Severo M, Fátima Pina M, et al. Sociodemographic determinants of prevalence and incidence of Helicobacter pylori infection in Portuguese adults. Helicobacter. 2013;18(6):413-22.

Bettencourt P, Lourenço P, Azevedo A. Influence of socioeconomic status on therapy and prognosis after an acute heart failure episode. International journal of cardiology. 2013;168(5): 4985-4987.

Bingham DD, Varela-Silva MI, Ferrão MM, Augusta G, Mourão MI, Nogueira H, et al. Socio-demographic and behavioral risk factors associated with the high prevalence of overweight and obesity in portuguese children. American Journal of Human Biology. 2013;25(6):733-42.

Borrell C, Marí-Dell'olmo M, Palència L, Gotsens M, Burström BO, Domínguez-Berjón F, et al. Socioeconomic inequalities in mortality in 16 European cities. Scandinavian journal of public health. 2014;42(3): 245-254.

Bulhões C, Ramos E, Lindert J, Dias S, Barros H. Depressive symptoms and its associated factors in 13-year-old urban adolescents. International journal of environmental research and public health. 2013;10(10):5026-38.

Camões M, Lopes C, Oliveira A, Santos AC, Barros H. Overall and central obesity incidence in an urban Portuguese population. Preventive medicine. 2010;50(1):50-5.

Campos-Matos I, Peralta-Santos A, Gomes B, Borges G, Aguiar P. Body Mass Index Assessment of Health Care Professionals in a Primary Care Setting in Portugal: a Cross Sectional Study. Acta Médica Portuguesa. 2014;27(5):609-614.

Carvalho J, Nobre P. Gender issues and sexual desire: The role of emotional and relationship variables. The journal of sexual medicine. 2010;7(7):2469-78.

Carvalho M, Matos MG. Psychosocial Determinants of Mental Health and Risk Behaviours in Adolescents. Global journal of health science. 2014;6(4):p22.

Correia S, Rodrigues T, Barros H. Socioeconomic variations in female fertility impairment: a study in a cohort of Portuguese mothers. BMJ open. 2014;4(1):e003985.

Correia S, Barros H. Small-for-gestational age Portuguese babies: the effect of childhood social environment, growth and adult socioeconomic conditions. Preventive medicine. 2015;70:102-7.

Costa C, Ramos E, Severo M, Barros H, Lopes C. Determinants of eating disorders symptomatology in Portuguese adolescents. Archives of pediatrics & adolescent medicine. 2008;162(12):1126-32.

Dias S, Gama A, Martins MO. Health status and preventative behaviors of immigrants by gender and origin: A Portuguese cross-sectional study. Nursing & health sciences. 2013;15(3):309-17.

Eikemo TA, Huisman M, Bambra C, Kunst AE. Health inequalities according to educational level in different welfare regimes: a comparison of 23 European countries. Sociology of health & illness. 2008;30(4):565-82.

Falcão H, Ramos E, Marques A, Barros H. Prevalence of asthma and rhinitis in 13 year old adolescents in Porto, Portugal. Revista Portuguesa de Pneumologia (English Edition). 2008;14(6):747-768.

Ferrão MM, Gama A, Marques VR, Mendes LL, Mourão I, Nogueira H, Velásquez-Melendez G, Padez C. Association between parental perceptions of residential

neighbourhood environments and childhood obesity in Porto, Portugal. European journal of public health. 2013;23(6):1027-1031.

Ferreira-Pinto LM, Rocha-Gonçalves F, Teixeira-Pinto A. An ecological study on the geographic patterns of ischaemic heart disease in Portugal and its association with demography, economic factors and health resources distribution. BMJ open. 2012;2(4):e000595.

Ferreira-Valente MA, Pais-Ribeiro JL, Jensen MP. Associations between psychosocial factors and pain intensity, physical functioning, and psychological functioning in patients with chronic pain: A cross-cultural comparison. The Clinical journal of pain. 2014;30(8):713-23.

Fraga S, Marques-Vidal P, Vollenweider P, Waeber G, Guessous I, Paccaud F, et al. Association of socioeconomic status with inflammatory markers: A two cohort comparison. Preventive medicine. 2015;71:12-19.

Gotsens M, Marí-Dell'Olmo M, Pérez K, Palència L, Martinez-Beneito MA, Rodríguez-Sanz M, Burström B et al. Socioeconomic inequalities in injury mortality in small areas of 15 European cities. Health & place. 2013;24:165-172.

Goulão B, Santos O, Alarcão V, Portugal R, Carreira M, do Carmo I. Prevalência de excesso de peso nos imigrantes brasileiros e africanos residentes em Portugal. Revista Portuguesa de Saúde Pública. 2015;33(1):24-32.

Harding S, Santana P, Cruickshank JK, Boroujerdi M. Birth weights of black African babies of migrant and nonmigrant mothers compared with those of babies of European mothers in Portugal. Annals of epidemiology. 2006a;16(7):572-9.

Harding S, Boroujerdi M, Santana P, Cruickshank J. Decline in, and lack of difference between, average birth weights among African and Portuguese babies in Portugal. International journal of epidemiology. 2006b;35(2):270-6.

Harding S, Teyhan A, Rosato M, Santana P. All cause and cardiovascular mortality in African migrants living in Portugal: evidence of large social inequalities. European Journal of Cardiovascular Prevention & Rehabilitation. 2008;15(6):670-6.

Hoffmann R, Borsboom G, Saez M, Dell'Olmo MM, Burstrom B, Corman D, et al. Social differences in avoidable mortality between small areas of 15 European cities: an ecological study. Int J Health Geogr. 2014;13(8).

Humboldt S, Leal I, Pimenta F. Living Well in Later Life: The Influence of Sense of Coherence, and Socio-Demographic, Lifestyle and Health-Related Factors on Older Adults' Satisfaction with Life. Applied Research in Quality of Life. 2014;9(3):631-42.

Knesebeck O, Verde PE, Dragano N. Education and health in 22 European countries. Social science & medicine. 2006;63(5):1344-51.

Lawlor DA, Harro M, Wedderkopp N, Andersen LB, Sardinha LB, Riddoch CJ, et al. Association of socioeconomic position with insulin resistance among children from Denmark, Estonia, and Portugal: cross sectional study. BMJ. 2005;331(7510):183.

Leurent B, Nazareth I, Bellón-Saameño J, Geerlings MI, Maaroos H, Saldivia S et al. Spiritual and religious beliefs as risk factors for the onset of major depression: an international cohort study. Psychological medicine. 2013;43(10):2109-2120.

Machado-Rodrigues AM, Coelho-e-Silva MJ, Mota J, Cumming SP, Riddoch C, Malina RM. Correlates of aerobic fitness in urban and rural Portuguese adolescents. Annals of human biology. 2011;38(4):479-484.

Machado-Rodrigues AM, Coelho-E-Silva MJ, Mota J, Padez C, Martins RA, Cumming SP et al. Urban–rural contrasts in fitness, physical activity, and sedentary behaviour in adolescents. Health promotion international. 2012;29(1):118-29.

Machado-Rodrigues AM, Santana A, Gama A, Mourão I, Nogueira H, Rosado V, Padez C. Parental perceptions of neighborhood environments, BMI, and active behaviors in girls aged 7–9 years. American Journal of Human Biology. 2014; 26(5):670-675.

Malmusi D. (2014). Immigrants' health and health inequality by type of integration policies in European countries. The European Journal of Public Health. 2014;25(2):293-9.

Marí-Dell'Olmo M, Gotsens M, Palència L, Burström B, Corman D, Costa G et al. Socioeconomic inequalities in cause-specific mortality in 15 European cities. Journal of epidemiology and community health. 2015;69(5):432-441.

Martins IP, Maruta C, Freitas V, Mares I. Executive performance in older Portuguese adults with low education. The Clinical Neuropsychologist. 2013;27(3):410-425.

Mastekaasa A. The gender gap in sickness absence: long-term trends in eight European countries. The European Journal of Public Health. 2014;cku075.

Mello T, Antunes I, Waldman E, Ramos E, Relvas M, Barros H. Prevalence and severity of dental caries in schoolchildren of Porto, Portugal. Community dental health. 2008;25(2):119-25.

Miranda MJ, Costa C, Santana P, Barrozo LV. Associação espacial entre variáveis socioeconômicas e risco relativo de nascimentos pré-termo na Região Metropolitana de São Paulo (RMSP) e na Área Metropolitana de Lisboa (AML). Saúde Soc. 2014;23(4):1142-53.

Neto F. Predictors of mental health among adolescents from immigrant families in Portugal. Journal of Family Psychology. 2009;23(3):375.

Neto F. Mental health among adolescents from returned Portuguese immigrant families from North America. North American Journal of Psychology. 2010;12(2):265.

Nogueira H, Ferrão M, Gama A, Mourão I, Marques VR, Padez C. Perceptions of neighborhood environments and childhood obesity: evidence of harmful gender inequities among Portuguese children. Health & place. 2013a;19:69-73.

Nogueira H, Gama A, Mourao I, Marques V, Ferrão M, Padez C. The associations of SES, obesity, sport activity, and perceived neighborhood environments: Is there a model of environmental injustice penalizing portuguese children? American journal of human biology. 2013b;25(3):434-436.

Nunes B, Silva RD, Cruz VT, Roriz JM, Pais J, Silva MC. Prevalence and pattern of cognitive impairment in rural and urban populations from Northern Portugal. BMC neurology. 2013;10(1):42.

Oliveira LDP, Pereira ML, Azevedo A, Lunet N. Risk factors for cardiovascular disease among the homeless and in the general population of the city of Porto, Portugal. Cadernos de Saúde Pública. 2012;28(8):1517-1529.

Oliveira C, Economou T, Bailey T, Mendonça D, Pina M. The interactions between municipal socioeconomic status and age on hip fracture risk. Osteoporosis International. 2015;26(2):489-98.

Pereira M, Canavarro MC. Gender and age differences in quality of life and the impact of psychopathological symptoms among HIV-infected patients. AIDS and Behavior. 2011;15(8):1857-69.

Pereira M, Oliveira L, Lunet N. Caries and oral health related behaviours among homeless adults from Porto, Portugal. Oral health & preventive dentistry. 2013;12(2):109-116.

Perelman J, Fernandes A, Mateus C. Gender disparities in health and healthcare: results from the Portuguese National Health Interview Survey. Cadernos de saude publica. 2012;28(12):2339-48.

Perelman J. Are chronic diseases related to height? Results from the Portuguese National Health Interview Survey. Economics & Human Biology. 2014;15:56-66.

Pimenta F, Leal I, Maroco J, Ramos C. Perceived control, lifestyle, health, socio-demographic factors and menopause: Impact on hot flashes and night sweats. Maturitas. 2011;69(4):338-42.

Ramos, E, Barros H. Family and school determinants of overweight in 13-year-old Portuguese adolescents. Acta Paediatrica. 2007;96(2): 281-286.

Ribeiro O, Teixeira L, Araújo L, Afonso RM, Pachana N. Predictors of anxiety in centenarians: health, economic factors, and loneliness. International Psychogeriatrics. 2014:1-10.

Rodrigues T, Barros H. Maternal unemployment: an indicator of spontaneous preterm delivery risk. European journal of epidemiology. 2008;23(10):689-693.

Ruiz M, Goldblatt P, Morrison J, Kukla L, Švancara J, Riitta-Järvelin M et al. Mother's education and the risk of preterm and small for gestational age birth: a DRIVERS meta-analysis of 12 European cohorts. Journal of epidemiology and community health. 2015 jech-2014.

Santana P, Costa C, Loureiro A, Raposo J, Boavida JM. Geografias da diabetes mellitus em Portugal: como as condições do contexto influenciam o risco de morrer. Acta Med Port. 2014;27(3):309-17.

Santos AC, Barros H. Prevalence and determinants of obesity in an urban sample of Portuguese adults. Public Health. 2003;117(6):430-7.

Santos AC, Ebrahim S, Barros H. Gender, socio-economic status and metabolic syndrome in middle-aged and old adults. BMC Public Health. 2008;8(1):62.

Santos AC, Severo M, Barros H. Incidence and risk factors for the metabolic syndrome in an urban South European population. Preventive medicine. 2010;50(3):99-105.

Santos DA, Silva AM, Santa-Clara H, Matias CN, Fields DA, Sardinha LB. Determinant factors of cardiorespiratory fitness in Portuguese adolescents of different ethnicities. Revista Brasileira de Cineantropometria & Desempenho Humano. 2011;13(4):243-9.

Santos NC, Costa PS, Cunha P, Portugal-Nunes C, Amorim L, Cotter J et al. Clinical, physical and lifestyle variables and relationship with cognition and mood in aging: a cross-sectional analysis of distinct educational groups. *Frontiers in aging neuroscience. 2014a;6:21*.

Santos R, Moreira C, Abreu S, Lopes L, Ruiz JR, Moreira P et al. Parental Education Level Is Associated With Clustering of Metabolic Risk Factors in Adolescents Independently of Cardiorespiratory Fitness, Adherence to the Mediterranean Diet, or Pubertal Stage. *Pediatric cardiology*. 2014b;*35*(6):959-964.

Schütte S, Chastang JF, Parent-Thirion A, Vermeylen G, Niedhammer I. Social differences in self-reported health among men and women in 31 countries in Europe. Scandinavian journal of public health. 2013;41(1):51-57.

Silva PA. Individual and social determinants of self-rated health and well-being in the elderly population of Portugal. Cadernos de saude publica. 2014;30(11):2387-400.

Sousa-Ribeiro M, Sverke M, Coimbra JL. Perceived quality of the psychosocial environment and well-being in employed and unemployed older adults: the importance of latent benefits and environmental vitamins. Economic and Industrial Democracy. 2014;35(4):629-52.

Stewart-Knox B, Duffy ME, Bunting B, Parr H, de Almeida MDV, Gibney M. Associations between obesity (BMI and waist circumference) and socio-demographic factors, physical activity, dietary habits, life events, resilience, mood, perceived stress and hopelessness in healthy older Europeans. BMC public health. 2012;12(1):424.

Vilhena E, Pais-Ribeiro J, Silva I, Pedro L, Meneses RF, Cardoso H et al. Psychosocial factors as predictors of quality of life in chronic portuguese patients. Health Qual Life Outcomes. 2014;12(1):3.

Williamson LM, Rosato M, Teyhan A, Santana P, Harding S. AIDS mortality in African migrants living in Portugal: evidence of large social inequalities. Sexually transmitted infections. 2009;85(6):427-31.
